# Tooth whitening with an experimental toothpaste containing hydroxyapatite nanoparticles

**DOI:** 10.1186/s12903-022-02266-3

**Published:** 2022-08-08

**Authors:** Ren Shang, Dalia Kaisarly, Karl-Heinz Kunzelmann

**Affiliations:** Department of Conservative Dentistry and Periodontology, University Hospital, LMU Munich, Goethestrasse 70, D-80336 Munich, Germany

**Keywords:** Toothpaste, Hydroxyapatite, Nanotechnology, Whitening, Concentration

## Abstract

**Background:**

The aim of this study was to evaluate the postbrushing tooth-whitening effect of toothpaste containing hydroxyapatite nanoparticles (nano-HAPs). The impact of the concentration on the whitening performance of nano-HAP toothpaste was also investigated.

**Methods:**

Two concentrations of nano-HAP (10 wt% and 1 wt%) were incorporated in nonabrasive toothpastes. Forty bovine incisors were randomly assigned into four groups: 10 wt% nano-HAP, 1 wt% nano-HAP, toothpaste without nano-HAP as a negative control and water as a blank control. Each tooth was treated with the toothpaste three times and hydrodynamic shear force (HSF) once. The teeth surfaces were observed by SEM after each application. Tooth color (L*, a* and b* values) was measured by a spectrophotometer, and color changes (△*E*, △*L*, △*a* and △*b* values*)* were calculated. Two-way mixed ANOVA was performed to evaluate the influence of the concentration and repeated application on the tooth-whitening effect of nano-HAP.

**Results:**

We found that nano-HAP-treated enamel exhibited higher L* values and lower a* and b* values than the control groups (*P* < 0.05). The 10 wt% nano-HAP group showed significantly higher △*E* values than the 1 wt% nano-HAP group (*P* < 0.05). After three applications, the △*E* mean value of the 10 wt% nano-HAP group was 4.47. The △*E* and △*L* values were slightly reduced after HSF (*P* < 0.05). For both nano-HAP groups, HAP single crystallites and agglomerates were identified, and their sizes grew with nano-HAP reapplication.

**Conclusions:**

In conclusion, nano-HAP toothpaste has a satisfying postbrushing whitening effect and good resistance to mechanical forces. The whitening effect seemed to be concentration-dependent.

## Introduction

The color of the tooth is a complex optical phenomenon. When the incident light falls on the tooth surface, it undergoes a process of specular and diffuse reflection at the surface and absorption and scattering within the dental tissue [1, 2]. Any process that changes the tooth characteristics, such as gloss, curvature and texture, opacity and translucency, can affect the light reflection and thereby influence the tooth appearance [3]. For example, an age-related change in tooth color can occur due to a reduction in enamel thickness, which makes the teeth appear darker since the dentin shines through more. In addition to this physiological change, tooth color is also affected by a combination of intrinsic and extrinsic discoloration. Intrinsic discoloration, such as tetracycline teeth or dental fluorosis, is caused by the incorporation of chromophores into enamel and dentin, while extrinsic discoloration refers to superficial stains on the tooth surface, which are usually caused by the consumption of tobacco, coffee and tea. It was reported that 28% of adults in the United Kingdom, 34% of adults in the United States, and 56% of adults in China are dissatisfied with the appearance or color of their teeth [4, 5]. According to the American Academy of Cosmetic Dentistry, tooth whitening has become one of the most popular and common procedures in cosmetic dentistry [6].

Conventional over-the-counter (OTC) whitening approaches include physical removal of stains with abrasives and detergents and chemical degradation of chromophores with peroxide-based agents [7]. However, these all have potential adverse effects, such as abrasion, posttreatment hypersensitivity, mucosal irritation and genotoxicity. This limits their use in patients with worn enamel, erosion, exposed dentin, or during pregnancy [8, 9]. In addition, the EU Council Directive 2011/84/EU stated that OTC bleaching products that contain higher than 0.1% hydrogen peroxide cannot be sold to patients without the supervision of dentists [10]. Therefore, alternative nonoxidative tooth-whitening formulations are needed.

Alternative whitening products should not only be gentle and suitable for a considerable range of consumers but also efficient for daily at-home dental care. Hydroxyapatite (HAP) is considered one of the most biocompatible and biomimetic materials, since it has a high similarity to the apatite crystal of human tooth enamel in terms of its morphology and crystal structure [11, 12]. In addition to its wide application in preventive dentistry, oral surgery, periodontology and implantology [13, 14], the whitening effect of HAP has gradually attracted the attention of dental researchers.

In 2001, Niwa and colleagues [15] found that toothpaste containing HAP had a tooth-whitening effect, which was not caused by their polishing effect. Four years later, Yamagishi and colleagues [13] reported that HAP could adhere to the enamel surface after pretreatment with phosphoric acid, forming a layer that covers the entire tooth surface. This newly formed layer was then proven to be able to increase the reflection of light, which leads to a measurable enhancement in lightness [16]. In the following decade, some in vitro studies [17–20] confirmed the tooth-whitening effect of aqueous HAP suspensions, which could be used clinically as nonoxidizing whitening mouth rinses. However, the problem is that a patient has to buy an additional oral care product in the form of a mouth rinse to fulfill their desire for whiter teeth. To make it easier for patients, it would be nice if a HAP toothpaste exhibits similar tooth-whitening ability. This would allow a patient to achieve whitening and cleaning purposes with a single product. Recently, Vorleitner and colleagues [21] mixed HAP materials into a commercial toothpaste and found that the incorporation of HAP did not result in any tooth color changes. However, the interaction between the HAP material and the ingredients in the toothpaste are unknown because neither the ingredients nor their amount was reported. Some ingredients may influence the whitening performance of commercial toothpastes with HAP additives such as fluoride or metaphosphates.

To date, there is no consensus on the influence of the nano-HAP concentration on the tooth-whitening effect. A quantitative analysis confirmed a positive correlation between the HAP concentration and adhesion efficiency [22]. Thus, increasing the HAP concentration may positively influence its whitening outcome. However, this assumption was not observed by two previous studies, in which nano-HAP was mixed into different substrates (such as a dissolvable polymer film and a self-assembling peptide) to achieve an enhancement of adhesion [18, 19]. The unique spatial molecular structures of the substrates may interfere with revealing the impact of the HAP concentration on its whitening effect. Studies addressing the role of its concentration in the whitening process of nano-HAP toothpastes with known composition are still lacking.

To fill these gaps, we prepared nonabrasive nano-HAP toothpastes and applied them to the enamel surface with a real toothbrush to simulate the daily toothbrushing process. Our objective was to evaluate the potential of self-administered use of HAP toothpaste. The null hypothesis states that the HAP concentration does not influence the whitening effect of HAP toothpaste.

## Materials and methods

### Preparation of toothpaste slurries

Abrasive-free toothpaste slurries were prepared as described by Wiegand and colleagues [23]. Briefly, the toothpaste slurries consisted of artificial saliva (Pharmacy of the LMU Munich, Germany), glycerine, sodium bicarbonate, and carboxymethylcellulose. Nano-HAP (NanoXIM CarePaste, Fluidinova, Portugal) was then added to prepare the toothpaste slurries, which contained two different concentrations of nano-HAP (1 wt% and 10 wt%). The toothpaste slurries were stirred before each application to ensure the homogeneity of the nano-HAP.

### Preparation of tooth samples

**Fig. 1:**
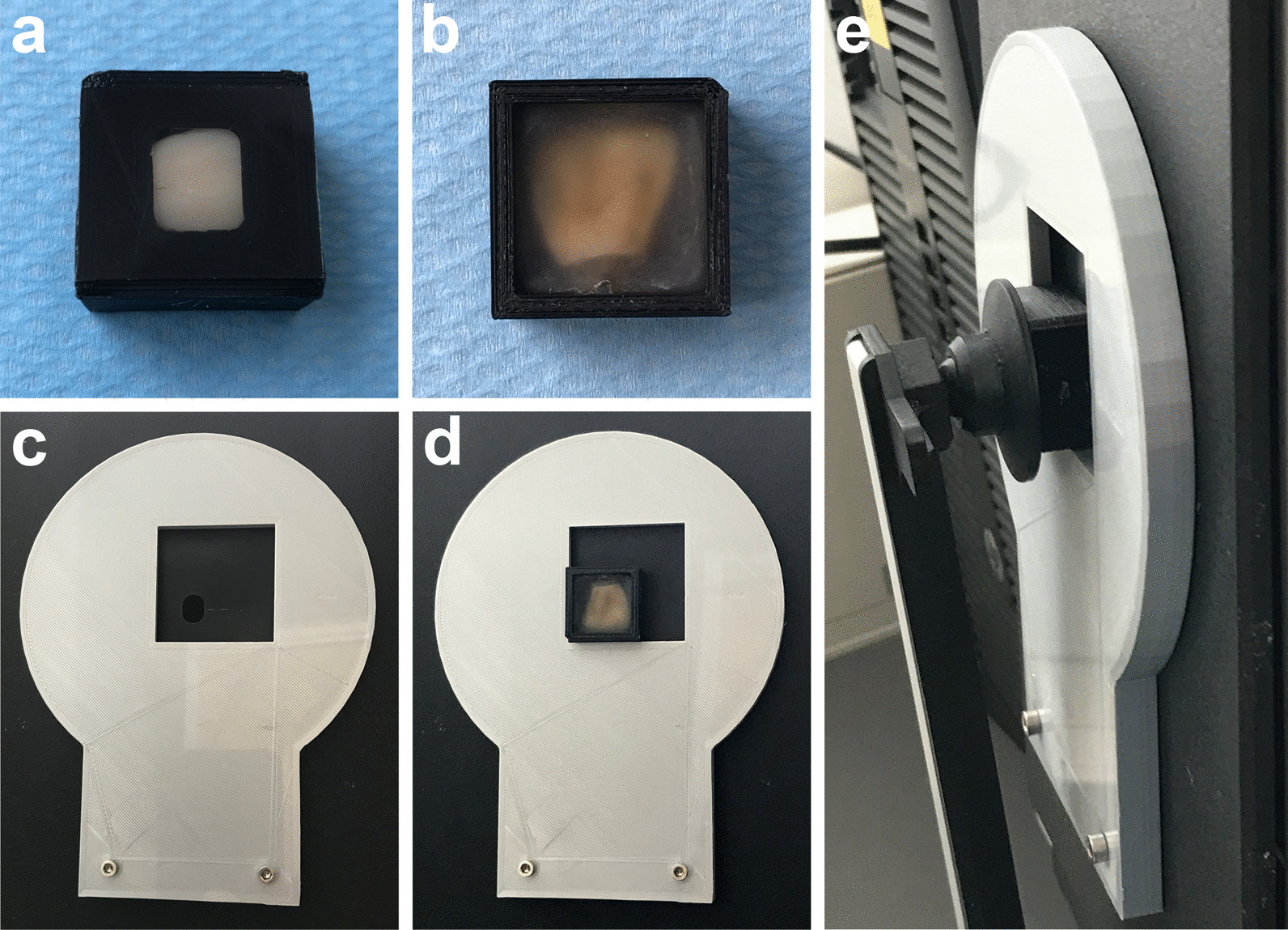
The 3D-printed repositioning system for the color measurement. A bovine tooth was embedded in the sample holder (**a** and **b**). The size of the measuring window of the sample holder is identical to that of the spectrophotometer (**c**). The embedded tooth was placed on the lower-left corner of the position locator (**d**), which was fixed on the spectrophotometer. The sample was held firmly by the movable arm during the color measurement (**e**)

Only bovine teeth that had intact enamel surfaces were included in this study. After careful removal of connective tissue from the bovine teeth, the teeth were rinsed with distilled water. Then, they were stained artificially. The staining solution was prepared by dissolving six grams of instant coffee (Nescafe Espresso, Nestle AG, Germany) in 200 ml of boiling water. The coffee solution was centrifuged at 2000 × g/min for 10 min (ROTIXA/A, Hettlich, Germany). The tooth samples were stored in the supernatant at 37 °C for three days. The samples were then polished with a polishing paste and ultrasonically cleaned for 10 min to remove all extrinsic stains. The teeth that were darker than A3 were embedded into 3D-printed specimen holders using self-curing polyester material (Technovit 4000, Kulzer Technik, Germany) (Fig. 1a and b). The specimen holders had a measurement window that allowed color measurements to be made on the middle third of the labial enamel surface. Only the teeth, whose color was stable over three consecutive measurements (△*L*, △*a* and △*b* values between every two consecutive measurements were lower than 0.05) were chosen for further study.

### Application of toothpaste slurry

A total of 40 tooth samples were randomly assigned to four groups: toothpaste slurry with 10 wt% nano-HAP (*n* = 10), toothpaste slurry with 1 wt% nano-HAP (*n* = 10), toothpaste slurry without nano-HAP as a negative control (*n* = 10), and water as a blank control (*n* = 10). A manual toothbrushing with a commercial electric toothbrush (IO, Oral B, Germany) was applied. The speed of brushing was 60 brushing cycles per minute. The duration of toothbrushing was 30 s, which referred to a speed of 30 times of a complete movement of the brush-head in a back-and-forth or circular direction in 30 s. The brushing force was controlled between 0.8 and 2.5 N. The brushing angulation was 90°. A combination of vertical and rotational movement with a back-and-force length of 2.5 cm was applied.

After brushing, the samples were gently rinsed with distilled water and stored in artificial saliva at 37 °C for 24 h before the next application. We applied the nano-HAP three times to the tooth surfaces, which were described as HAP 1, HAP 2 and HAP 3.

### Application of hydrodynamic shear force

To evaluate the mechanical stability of the interaction between adhered HAP particles and the enamel surface, a hydrodynamic shear force (HSF) was generated by an electric toothbrush and applied to each sample for 2 min to simulate mechanical forces in the patient’s mouth after HAP 3. The tips of the bristle were mounted at 1 mm from the enamel surfaces. The bristle motion of the toothbrush generated a turbulent fluid flow that directly caused a hydrodynamic effect on the tooth surfaces [24].

### Color measurement

The color of each tooth was measured with a spectrophotometer (Color-Eye 7000, Gretag MacBeth X-Rite-, Germany). To ensure the repositioning and reproducibility of the color measurement, the samples were placed on the lower-left corner of a 3D-printed position locator, which was fixed firmly to the spectrophotometer (Fig. 1c, d and e), and the measurements were performed in darkness. To avoid enamel dehydration, we placed a cover glass on the measuring side of the embedded samples and added a few drops of water between the tooth and the cover glass. The color assessment was defined by the Commission Internationale de I’Eclairage (CIE) LAB color system. The baseline color of the teeth (*L*_*0*_, *a*_*0*_ and *b*_*0*_) was obtained before all applications, and the color (*L*, *a* and *b*) at each experimental time point (HAP 1–3 and HSF) was measured 24 h after each application. The average color changes (△*E* values) were determined as the Euclidean distance of the measuring points in the three-dimensional Lab color space $\varDelta E=\sqrt{{\left(\varDelta L\right)}^{2}+{\left(\varDelta a\right)}^{2}+{\left(\varDelta b\right)}^{2}, }{\varDelta L=L}_{}-{L}_{0}$, ${\varDelta a=a}_{}-{a}_{0}$, ${\varDelta b=b}_{}-{b}_{0}$.

### SEM evaluation

**Fig. 2:**
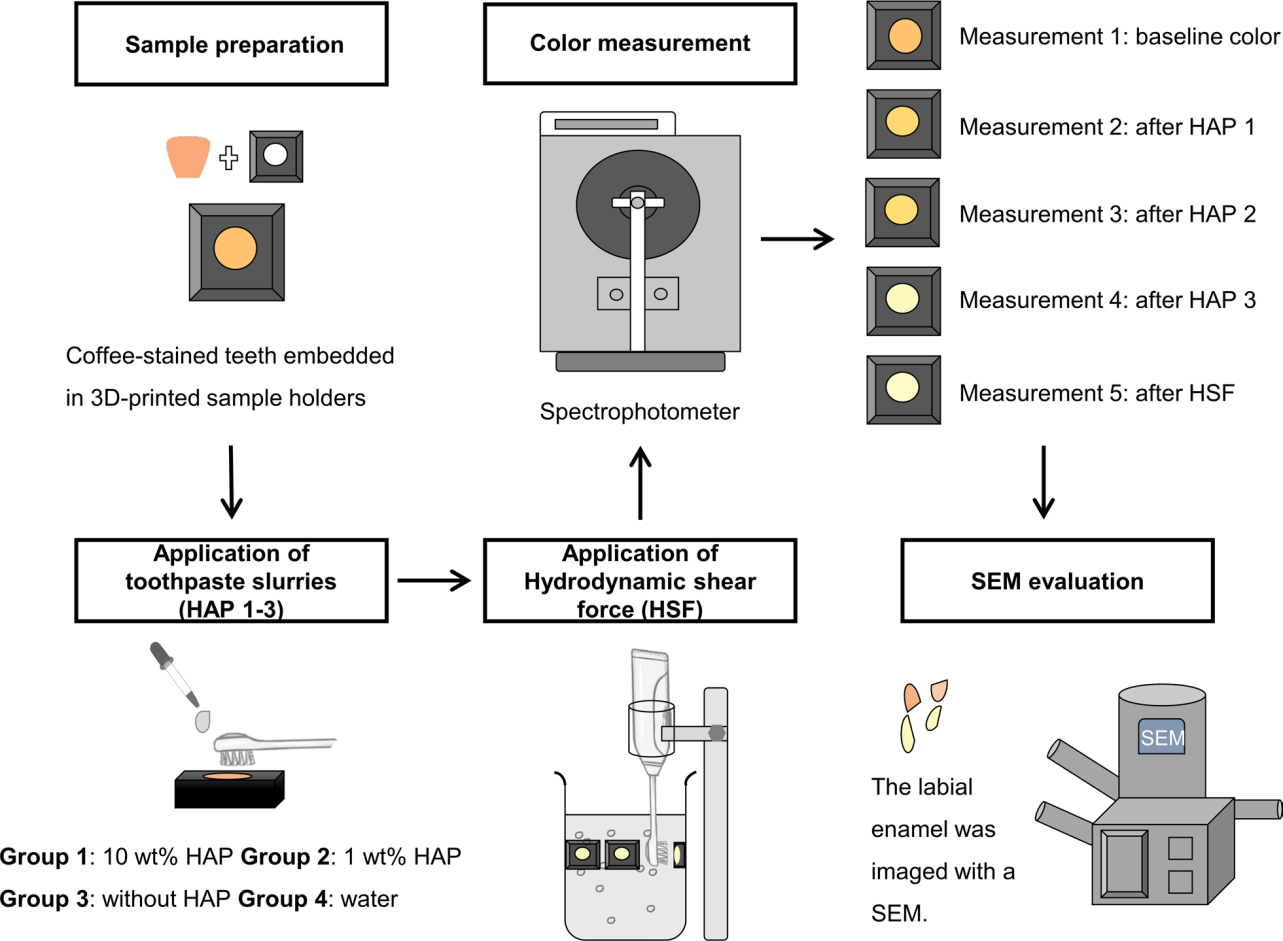
The workflow of the present study

Sixteen additional bovine incisors were again randomly selected and assigned to the four groups (*n* = 4 for each group). Nano-HAP toothpaste and HSF were applied as described above. The samples were cut along the median line into two parts. The labial part was chosen for SEM evaluation. After dehydration in increasing concentrations of ethanol, the specimens were sputter-coated with a thin (25 nm) layer of a gold-palladium alloy (SC 7620, Polaron, Quorum Technologies, Kent, UK). Subsequently, the sample was imaged with a field-emission scanning electron microscope (FE-SEM, Supra 55 vp, Zeiss, Germany) at a magnification of 5000 ×. The workflow of the present study is shown in Fig. 2.

### Statistical analysis

The data were analyzed in R. All data were tested for normality (Shapiro-Wilk test), homoscedasticity (Levene’s test) and homogeneity of covariance (Box’s M-test). Two-way mixed analysis of variance (ANOVA) was performed to determine the effects of the concentration and repeated application on the tooth-whitening effect of nano-HAP. A Box–Cox transformation was applied to the data before the ANOVA test to meet all of the statistical assumptions. Tukey post hoc pairwise comparisons were used to compare individual groups. Paired t-tests were employed to investigate the impact of the hydrodynamic condition on the whitening effect of nano-HAP, where HAP 3 and HSF were compared. Statistical significance was identified at *P* < 0.05.

Power analysis was performed by software G*Power 3 software [25] to evaluate whether the evidence of the small sample size was strong enough to detect the whitening effect of nano-HAP toothpaste.

## Results

### Influence of the HAP concentration and applications

The results of the two-way mixed ANOVA are shown in Table 1. A significant main effect of HAP concentration was found on the △*E*, △*L*, △*a* and △*b* values (*P* < 0.05). A significant main effect of applications on the △*E*, △*a* and △*b* values was also observed (*P* < 0.05). No significant interaction between the two main effects was found (*P* > 0.05). The power analysis showed 100% power of the data evidence.

**Table 1:** Two-way mixed ANOVA analysis of the two main factors of HAP concentration and applications on the tooth-whitening effect of nano-HAP toothpaste

Factor	Sum of squares	DF	Mean square	F
*ΔE*				
HAP concentration	38.71	2.00	19.35	37.96^*^
Applications	4.86	1.37	3.56	34.74^*^
Interaction	0.28	2.73	0.10	1.01
*ΔL*				
HAP concentration	27.68	2.00	13.84	20.60^*^
Applications	0.21	1.43	0.15	1.07
Interaction	0.35	2.86	0.12	0.87
*Δa*				
HAP concentration	260.24	2.00	130.12	4.22^*^
Applications	37.36	1.39	26.96	5.39^*^
Interaction	37.92	2.77	13.68	2.74
*Δb*				
HAP concentration	879.38	2	439.69	14.41^*^
Applications	12.64	1.05	11.99	1.77
Interaction	54.32	2.11	25.75	3.80^*^

* P < 0.05

**Fig. 3:**
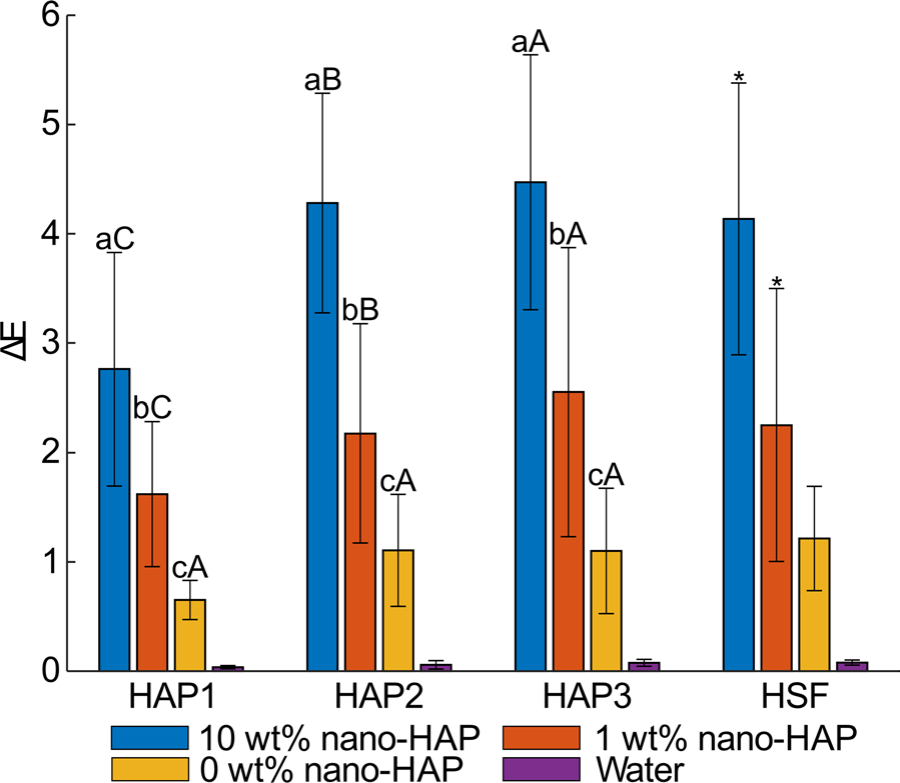
The average color changes (expressed as △*E* values) at each experimental time point (HAP 1: the first circle of nano-HAP application; HAP 2: the second circle of nano-HAP application; HAP 3: the third circle of nano-HAP application; HSF: application of hydrodynamic shear force). Lowercase letters indicate the differences between different groups at the same experiment time point. Capital letters indicate the differences of the same group between different experiment time points. Different superscripts indicate groups that statistically differed from any other group (*P* < 0.05). Same superscripts suggest that groups showed no significant differences (*P* > 0.05). From a (A) to c (C), the mean value is decreased. * indicates the significant differences between HAP 3 and HSF (*P* < 0.05)

The △*E* values of each group are shown in Fig. 3. The 10 wt% nano-HAP group showed the highest △*E* mean values throughout the observation period, followed by the 1 wt% nano-HAP group, the tooth slurry group without nano-HAP (negative control group), and then the water group. This finding suggested a positive association between the HAP concentration and the whitening effect. A Tukey post hoc test revealed significant pairwise differences (*P* < 0.05) in the △*E* values between the 10 and 1 wt% nano-HAP groups (HAP 1–3), between the 10 wt% nano-HAP and negative control groups (HAP 1–3), and between the 1 wt% nano-HAP and negative control groups (HAP 2 and 3). With nano-HAP applications, the △*E* mean values of the 10 wt% nano-HAP group increased from 2.76 (HAP 1) to 4.28 (HAP 2) and then to 4.47 (HAP 3), whereas those values changed from 1.62 (HAP 1) to 2.17 (HAP 2) and then to 2.55 (HAP 3) in the 1 wt% nano-HAP group. The △*E* values increased statistically with HAP applications (*P* < 0.05), which indicated that repeated applications of HAP toothpaste could lead to more obvious tooth color changes.

The increased △*E* values of both nano-HAP groups were contributed by the increased L* values and decreased a* and b* values. The △*L*, △*a*, and △*b* values of each group are shown in Table 2. The 10 wt% nano-HAP group showed statistically higher △*L* values than the 1 wt% nano-HAP group after HAP 1 and 2 (*P* < 0.05). Meanwhile, the 10 wt% nano-HAP group exhibited statistically lower *△b* values than the 1 wt% nano-HAP group (HAP 2 and 3). This finding suggested that the higher concentration of nano-HAP changed the tooth color to a lighter and bluer tone than the lower concentration.

**Table 2:** The color changes in L*, a* and b* axes (expressed as Δ*L*, *Δa*, and *Δb* values) at each experimental time point (HAP 1–3, HSF)

Group	Experimental time point			
	HAP 1	HAP 2	HAP 3	HSF
*ΔL*				
10 wt% nano-HAP	2.30 (1.12)^aA^	2.70 (1.12)^aA^	2.76 (1.36)^aA^	2.09 (1.72)^*^
1 wt% nano-HAP	1.13 (0.73)^bB^	1.51 (1.20)^bAB^	1.78 (1.70)^aA^	1.30 (1.67)^*^
Without HAP	− 0.01 (0.42)^bA^	− 0.13 (0.68)^cA^	− 0.08 (0.73)^bA^	− 0.14 (0.87)
Water	0.01 (0.01)	0.01 (0.02)	0.00 (0.04)	0.00 (0.03)
*Δa*				
10 wt% nano-HAP	− 0.21 (0.21)^aA^	− 0.75 (0.90)^bB^	− 0.95 (0.86)^bB^	− 0.82 (0.63)
1 wt% nano-HAP	0.05 (0.47)^aA^	− 0.20 (0.72)^abA^	− 0.31 (0.83)^abA^	− 0.25 (0.85)
Without HAP	0.05 (0.33)^aA^	0.09 (0.40)^aA^	0.12 (0.36)^aA^	0.12 (0.37)
Water	0.01 (0.02)	− 0.04 (0.04)	− 0.03 (0.06)	− 0.03 (0.05)
*Δb*				
10 wt% nano-HAP	− 1.34 (0.64)^bA^	− 2.55 (1.84)^bB^	− 2.66 (1.90)^cB^	− 2.71 (1.86)
1 wt% nano-HAP	− 0.84 (0.63)^abA^	− 0.96 (0.83)^aA^	− 0.10 (0.83)^bA^	− 0.88 (0.94)
Without HAP	0.03 (0.47)^aA^	0.21 (0.96)^aA^	0.25 (0.96)^aA^	0.25 (0.93)
Water	0.00 (0.03)	− 0.01 (0.04)	0.02 (0.04)	0.01 (0.05)

Results are shown as mean value (standard deviation)

HAP 1: the first circle of nano-HAP application;

HAP 2: the second circle of nano-HAP application;

HAP 3: the third circle of nano-HAP application;

HSF: application of hydrodynamic shear force;

Lowercase letters indicate the differences between different groups at same experiment time point;

Capital letters indicate the differences of the same group between different experiment time points;

Different superscripts of individual letters indicate groups that statistically differed from any other group (*P* < 0.05);

Same superscripts suggest that groups showed no significant differences (*P* > 0.05);

From a (A) to c (C), the mean value is decreased;

* indicates the significant differences between HAP 3 and HSF (*P* < 0.05)

### Influence of the hydrodynamic condition

The tooth color changes caused by the application of hydrodynamic shear force were compared with those after HAP 3. For both nano-HAP groups, the HSF led to statistical reductions in the △*E* and △*L* values (*P* < 0.05). The △*a* and △*b* values were not significantly influenced by the hydrodynamic effect (*P* > 0.05).

### SEM evaluation

**Fig. 4:**
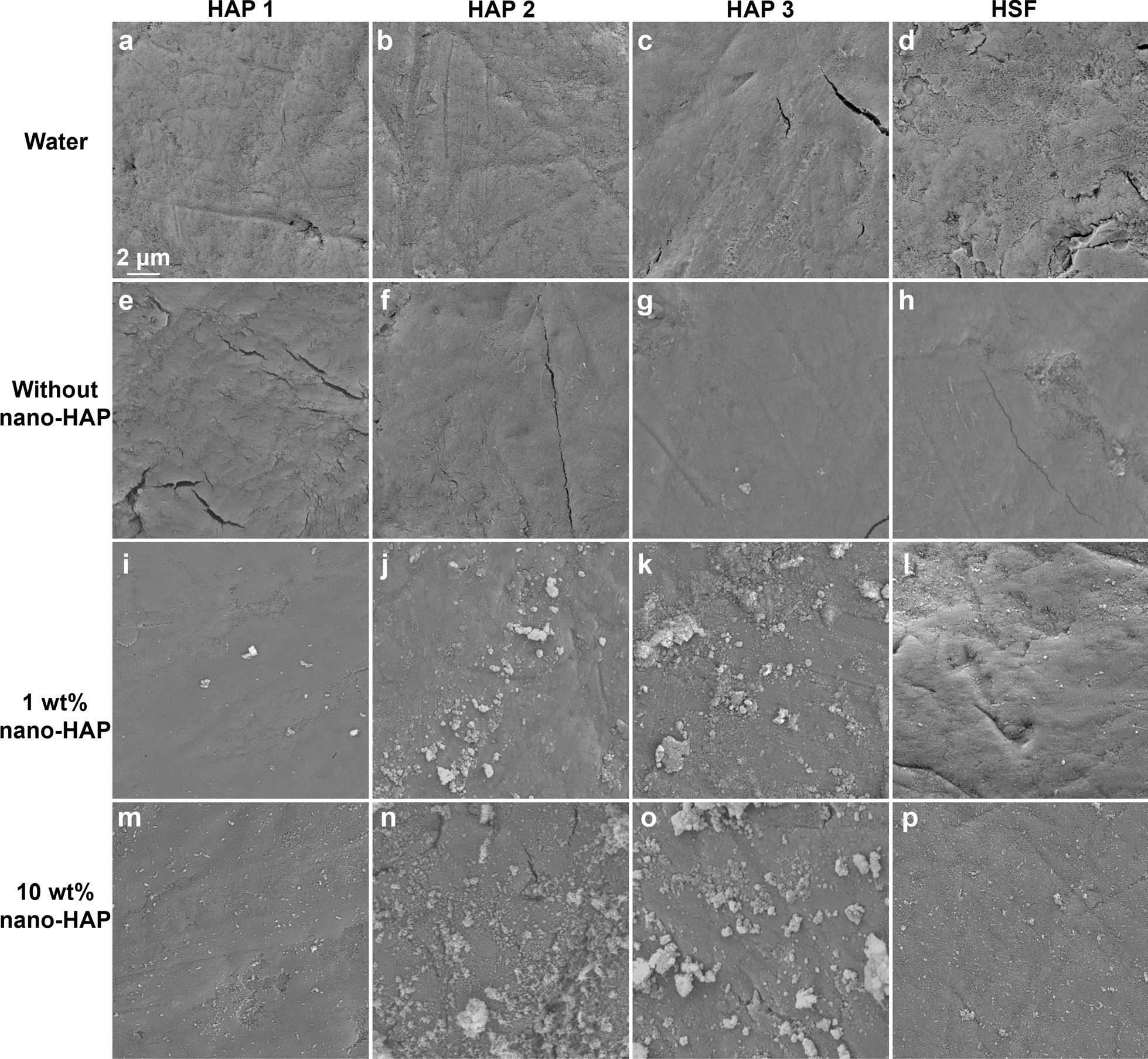
The nano-HAP adhesion on the enamel surface. **a**–**d**: water group; **e**–**h** toothpaste without nano-HAP; **i**–**l**: 1 wt% nano-HAP toothpaste; **m**–**p**: 10 wt% nano-HAP toothpaste. The sample was imaged with FE-SEM at the magnification of 5000 ×

The nano-HAP adhesion on the enamel surface is shown in Fig. 4. The photos of the two control groups exhibited the normal microstructure of enamel (Fig. 4a–h). For both nano-HAP groups, HAP single crystallites and agglomerates were observed throughout the experimental period (HAP 1–3, and HSF). After HAP 1, more nanosized agglomerates were identified in the 10 wt% nano-HAP group than in the 1 wt% nano-HAP group (Fig. 4i, m). After HAP 2 and 3, the nanosized crystallites and agglomerates grew larger and became microsized (Fig. 4j, k, n, o). Compared with HAP 1, more enamel surfaces were covered. Especially in the 10 wt% nano-HAP group, the tooth surface was almost completely covered. After HSF, most of the microsized agglomerates were removed, leaving the nanosized agglomerates to adhere relatively more firmly to the enamel surfaces (Fig. 4l, p).

## Discussion

The main goal of this study was to investigate the tooth-whitening effect of nano-HAP, used as an ingredient in toothpaste. Compared with the negative control group, the nano-HAP groups showed significantly higher △*E* values due to the increased L* values and the decreased a* and b* values, indicating that the nano-HAP toothpaste could make teeth appear brighter, less red and less yellow. These optical alterations can be explained by the increased diffuse light reflection and reduced light transmission through the tooth caused by the HAP particles adhering to the enamel [19, 26].

We applied the nano-HAP three times to the tooth surfaces and performed the color measurement 24 h after each application. This protocol was well accepted in previous studies, which focused on the whitening performance of HAP aqueous suspensions and a gel [17, 18, 27]. To compare the whitening performance of HAP toothpaste with these HAP formulations, this protocol was also used in the current study. After the three HAP applications, the △*E* mean values of the 10 wt% and the 1 wt% nano-HAP groups were 4.47 and 2.55, respectively. Considering that 50:50% perceptibility threshold of △*E* values in dentistry was shown to be 1.2 [28, 29], the color changes of both nano-HAP groups could be visually perceivable. The whitening performance of the 10 wt% nano-HAP toothpaste seemed better than that of a 44.4 wt% nano-HAP aqueous suspension, of which the △*E* value was 3.30 after the third use [18]. Several randomized clinical trials reported that the △*E* values of commercial abrasive toothpastes and peroxide-based toothpastes ranged from 2.25 to 4.46 with a clinical application period of up to 90 days [30, 31]. Therefore, it is considered that the 10 wt% nano-HAP toothpaste in our study had a satisfying postbrushing tooth-whitening effect. It is worth mentioning that the negative control group exhibited a slight whitening effect compared with the water group, with the mean values of △*E* fluctuating around approximately 1. The abrasive particles adhering to the enamel surface could also contribute to light scattering to some extent.

Understanding the relationship between the nano-HAP concentration and tooth-whitening effect is crucial for optimizing the efficiency of nano-HAP toothpaste. To analyze the role of the concentration more accurately, we did not make any structural modifications to the nano-HAP. To avoid interference from the ingredients of commercial toothpastes, none of them were incorporated into the toothpaste. Nano-HAP has been applied to some commercial toothpastes at concentrations up to 10 wt% [32], which was considered optimal for remineralization of early enamel caries [33]. Within this concentration, nano-HAP would not have any significant systemic exposure via the oral mucosa or cytotoxicity after a 48-hr exposure [32]. Therefore, a concentration of 10 wt% was also chosen as the upper limit in the present study.

The null hypothesis that the HAP concentration does not influence the whitening effect of HAP toothpaste could be rejected, as we found a significant main effect of the concentration on the △*E*, △*L*, △a, *a*nd △*b* values. Compared with the 1 wt% nano-HAP group, the 10 wt% nano-HAP group exhibited significantly higher △*E* values throughout the observation period. This finding could be explained by the SEM images. After HAP 1–3, more adhered HAP crystallites and agglomerates were observed in the 10 wt% nano-HAP group than in the 1 wt% group. The enamel coverage area was quantitatively calculated and shown to be increased from 10 to 30% with the HAP concentration increasing from 1 to 10 wt% [22]. More coverage may result in an increase in the reflection of light on the HAP layer and thereby lead to the increase in the △*L* values. This finding appeared to be well substantiated by a previous study [15], in which the increase in the amount of HAP in toothpaste resulted in the enhancement in the degree and rate of brightness. At the same time, more coverage could decrease the light transmission through enamel and dentin, which could lower the a* and b* values [19].

A significant main effect of the repeated application on the color changes was found in the current study. For both nano-HAP groups, the △*E* values increased significantly with the reapplication, which could be caused by the increased enamel coverage and the size change of the adherent particles from nanosized to microsized. These changes in the particle size appeared to be well substantiated by the previous studies, in which it was confirmed that the photoelectric characteristics and maturation time enabled the nano-HAP to be gathered within microsized conglomerates [34, 35]. A previous study reported that there was a maximal adhering load on enamel [17]. The tooth color does not change much when adhering saturation has been achieved. However, we did not find this saturation within three HAP reapplications. In our study, nano-HAP was applied to the enamel by tooth brushing. The loosely attached nano-HAP particles may be brushed away by mechanical friction.

From a clinical view of point, the adherent HAP agglomerates are exposed to mechanical forces in the patient’s mouth after brushing. HSF is often used to create force comparable to the mechanical stress caused by the movement of the lips and checks [36, 37]. For both nano-HAP groups, statistical reductions in the △*E* values were observed after the HSF application. Nevertheless, the △*E* mean value of the 10 wt% was still higher than 4, suggesting that a tooth whitening effect of the nano-HAP toothpaste is still to be expected under a loaded condition. After HSF application, most of the microsized agglomerates were removed from the enamel surfaces, while the nanosized agglomerates remained in place, which could be explained by their higher surface charges and stronger electrostatic forces [22, 34].

Our study has some particular strengths. First, we took a further step toward real clinical situations. We applied nano-HAP toothpaste to enamel by tooth brushing. The whitening effect of HAP toothpaste has been confirmed. Second, the interference factors were strictly controlled. For instance, no fluoride was added to the toothpaste slurries to avoid interaction between fluoride and HAP. Tooth dehydration could decrease enamel translucency and increase luminosity, making the tooth falsely appear whiter [38, 39]. Compared with previous studies [17, 18, 27], in which the samples were air-dried before the color measurement, we measured the tooth color in a liquid environment, which could avoid the interference of dehydration in tooth color assessment. Accurate repositioning is a key factor for color measurement, as the teeth are multilayered, translucent and exhibit color transitions in all directions [40]. However, the previously published studies did not address how the repositioning was achieved in their work [16, 18, 19]. To bridge this information gap, we invented a repositioning system by using 3D printing technology to enable accurate color measurement (Fig. 1).

Within the framework of laboratory tests, one tries to select the conditions in such a way that they can be transferred as well as possible to the clinical situation. Ideally, the tests would be carried out with human teeth. However, due to the success of prevention and modern filling materials, hardly any teeth are extracted in industrialized nations today that can be used for laboratory examinations. Although bovine enamel is somewhat more porous than human enamel, its chemical composition and surface properties are identical to those of human teeth. Therefore, we used bovine teeth in our study.

In this study, we applied HAP toothpaste by using the manual toothbrushing method. The duration of toothbrushing was 30 s for each sample. Assuming that a person has 28 teeth and one daily toothbrushing at home takes two minutes, the duration of 30 s of toothbrushing corresponds to the duration of seven times of daily toothbrushing for each sample. We would like to simulate toothbrushing in real daily home care, where the brushing speed and force could not be so strictly controlled as a brushing machine. We kept the application speed and force in a narrow range to reduce the impact of those factors on the whitening effect. For instance, brushing force ranged from 0.8 to 2.5 N [41]. The sensor light changes colors according to brushing force, which helps to maintain consistent pressure in the optimal range. If the force is more than 2.5 N, the sensor light will turn red and the smart drive will change the current mode into another mode that has a lower power. Considering that the nano-HAP was added to abrasive-free toothpaste slurries instead of to abrasive ones, we believed that the influence of brushing force on the whitening effect of nano-HAP in abrasive-free toothpastes might be not so determining in comparison to that in abrasive toothpastes.

We used artificial saliva instead of human saliva. The statherin and proline-rich glycoproteins in human saliva could bond strongly to HAP and thereby might influence its tooth whitening effect [42]. Moreover, human saliva has high variability of individual factors and complexity of its components [43]. Therefore, human saliva was not chosen.

The findings of this study have to be seen in light of some limitations. First, although we confirmed for the first time the whitening effect of nano-HAP toothpaste and the significant main effect of its concentration on tooth color changes, the whitening effect of nano-HAP after a prolonged application period needs to be further proven. Second, our in vitro study cannot reflect the complexity of the oral environment. Oral pH fluctuations and temperature changes also influence nano-HAP adhesion behavior [44].

## Conclusions

Considering the limitations of this study, nano-HAP toothpaste showed a postbrushing whitening effect and good resistance to mechanical forces. It could be considered a promising alternative to over-the-counter oxidizing bleaching products. The whitening effect is significantly affected by the concentration. Nano-HAP could be added to commercial toothpastes at a concentration of up to 10%, as this concentration showed visually perceptible tooth whitening performance in our study while being considered biologically safe.

## Data Availability

The data that support the findings of this study are available on request from the corresponding author.
